# Noise-Correlation Is Modulated by Reward Expectation in the Primary Motor Cortex Bilaterally During Manual and Observational Tasks in Primates

**DOI:** 10.3389/fnbeh.2020.541920

**Published:** 2020-12-02

**Authors:** Brittany Moore, Sheng Khang, Joseph Thachil Francis

**Affiliations:** ^1^Department of Biomedical Engineering, Cullen College of Engineering, The University of Houston, Houston, TX, United States; ^2^Department of Electrical and Computer Engineering, Cullen College of Engineering, The University of Houston, Houston, TX, United States

**Keywords:** motor cortex, reward, reinforcement learning, temporal difference, reward prediction, reward prediction error

## Abstract

Reward modulation is represented in the motor cortex (M1) and could be used to implement more accurate decoding models to improve brain-computer interfaces (BCIs; Zhao et al., 2018). Analyzing trial-to-trial noise-correlations between neural units in the presence of rewarding (R) and non-rewarding (NR) stimuli adds to our understanding of cortical network dynamics. We utilized Pearson’s correlation coefficient to measure shared variability between simultaneously recorded units (32–112) and found significantly higher noise-correlation and positive correlation between the populations’ signal- and noise-correlation during NR trials as compared to R trials. This pattern is evident in data from two non-human primates (NHPs) during single-target center out reaching tasks, both manual and action observation versions. We conducted a mean matched noise-correlation analysis to decouple known interactions between event-triggered firing rate changes and neural correlations. Isolated reward discriminatory units demonstrated stronger correlational changes than units unresponsive to reward firing rate modulation, however, the qualitative response was similar, indicating correlational changes within the network as a whole can serve as another information channel to be exploited by BCIs that track the underlying cortical state, such as reward expectation, or attentional modulation. Reward expectation and attention in return can be utilized with reinforcement learning (RL) towards autonomous BCI updating.

## Introduction

The neural representation of reward has been traced to deep brain structures including the substantia nigra pars compacta and ventral tegmental area (Schultz et al., 1997). Reward associated signals have also been shown to occur in multiple cortical areas including the orbitofrontal and sensorimotor cortices (Tremblay and Schultz, 1999; Marsh et al., 2015; McNiel et al., 2016a; Ramkumar et al., 2016; Ramakrishnan et al., 2017; An et al., 2018; Zhao et al., 2018). Previous studies have linked cortical responses to dopaminergic pathways, which can explain cortical responses on multiple time scales (Kunori et al., 2014) and indicates the complex interactions that exist between deep structure reward signaling and M1 units. For example, dopamine has proven necessary for learning in M1 (Molina-Luna et al., 2009) and has a significant effect on long-term potentiation (LTP) in the motor cortex, for review see Francis and Song (2011).

Studies of the motor cortex reveal a complex dynamical system (Shenoy et al., 2013). In addition to kinematic, directional (Georgopoulos et al., 1982), and force tuning information (Graham et al., 2003; Chhatbar and Francis, 2013), M1 neurons encode value and demonstrate reward modulation (Marsh et al., 2015; Ramkumar et al., 2016; Ramakrishnan et al., 2017; An et al., 2018, 2019; Zhao et al., 2018), as do regions associated with motor output (Platt and Glimcher, 1999; Musallam et al., 2004; Tanaka et al., 2004; Campos et al., 2005; Shuler and Bear, 2006; Louie et al., 2011; Toda et al., 2012; Zhao et al., 2018). Models indicate that signals encoding value and reward expectations in M1 play a significant role in the reinforcement learning (RL) process (Dura-Bernal et al., 2015; Tarigoppula et al., 2018). This information is encoded during active movement, as well as during action observation and motor planning which further implies an interconnection between reward, learning, and expectation (Tkach et al., 2007; Dushanova and Donoghue, 2010; Marsh et al., 2015).

Many of the single and multi-unit activity studies on reward modulation in M1 have utilized firing rate, with some exceptions (Marsh et al., 2015; An et al., 2018, 2019), where the local field potential (LFP) was studied. However, the correlation between individual units can also provide insight into how populations of neurons change with the task at hand (Maynard et al., 2011; Lee and Maunsell, 2009). There is notable variability in individual unit responses to repeated stimuli (Tolhurst et al., 1983), and this variation is often shared between neurons (Shadlen and Newsome, 1998). Research has shown that the correlation between trial-to-trial fluctuations has a significant impact on coding efficiency, specifically concerning attention, learning, and behavior (Zohary et al., 1994; Abbott and Dayan, 1999; Nirenberg and Latham, 2003; Averbeck and Lee, 2006; Cohen and Newsome, 2008).

Changes in correlation occur even in the absence of changes in firing rate, suggesting that correlation dynamics are a critical part of the neural code (Vaadia et al., 1995). Correlation reveals neural population interactions that contribute to our understanding of network architecture. Analysis of changes in correlation in different contexts indicates an increased correlation between similarly tuned ensembles and local circuits, affecting learning-related plasticity (Komiyama et al., 2010). Also, the correlation has proven useful in understanding potential connectivity across regions and populations (Reid and Alonso, 1995).

In studying the correlation of M1 units in the presence of cues associated with rewarding (R) and non-rewarding (NR) results, there are several factors to consider. Previous work has demonstrated correlation changes related to spatial attention, learning, and other aspects of the internal neural state. These factors are likely to coexist in a R task experimental paradigm with conditioned visual cues and may also be driving factors in our results. However, fluctuations in aspects of cortical state may occur on distinct and significantly different timescales, such as attentional shifts vs. circadian related changes. Second, correlation can be significantly affected by the strength of the stimulus-response (Ecker et al., 2010), which was shown to contribute to 33% of the across-study variance reported in V1 (Cohen and Kohn, 2011). Last, the composition of the population plays a role in the relationship between correlation and coding efficiency, which means that the results obtained may be interpreted differently depending on the ensemble of units with predictable variations across subpopulations. For example, while increasing correlation has proven detrimental in homogenous populations and similarly tuned neurons, it may increase information carrying capacity in heterogeneous populations and differently tuned units (Abbott and Dayan, 1999; Chelaru and Dragoi, 2008). As our task utilized a single target, we did not probe such relationships with tuning properties (see “Materials and Methods” section).

The goals of this study were to: (1) determine the extent and direction of changes in correlation in response to R and NR visual cues on M1 neural activity; and (2) interpret the role of correlation in improved neural encoding of R vs. NR trials. Given the capacity for reward modulation observed in previous studies within the sensorimotor cortices (Marsh et al., 2015; McNiel et al., 2016a,b; Ramkumar et al., 2016; Ramakrishnan et al., 2017; Zhao et al., 2018; An et al., 2019) and the association of decreased correlation with increased encoding accuracy, we predicted that the mean response of the M1 population would show noise-correlation decreases during R trials and that this decrease would include not only reward modulated units but the M1 network at large. We recently showed evidence in line with this hypothesis in the interaction between neural spike trains and the underlying LFP in M1 (An et al., 2019) with higher phase-amplitude coupling (PAC) and spike-field coherence (SFC) seen during the NR trials as compared to R trials, however, we did not report on interactions between single and multi-units, such as changes in the correlational structure between pairs of units as we do here.

## Materials and Methods

The data used in this study was originally collected and processed as described in Marsh et al. (2015).

### Experimental Task

**Figure 1:**
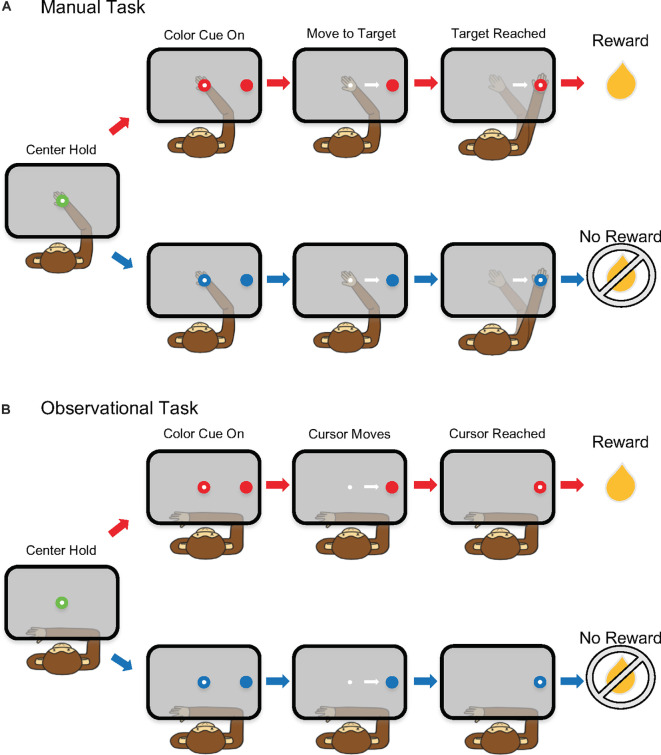
Schematic of the single target reaching task. **(A)** Manual task and **(B)** observational task (OT).

The non-human primates (NHPs) performed two tasks, manual and observational, that were used for analysis. First, the animals were trained to perform a single target center-out reaching task using their right arm inside a KINARM exoskeleton (BKIN Technologies). For the manual task, a right-hand movement was made from a central target to a peripheral target (0.8 cm radius) located 5 cm to the right (Figure 1). A cursor provided visual feedback of the hand position. The NHP initiated each trial by holding on the neutral (green) center target for 325 ms, followed by a variable color cue period (100–300 ms) where the colored peripheral target appeared as red of blue to indicate a R or NR trial, respectively. At the same time, the center target turned to the same color as the peripheral target. After a required hold period of 325–400 ms, a GO, marked by the disappearance of the center cue, indicated the NHP could move to the peripheral target where it had to hold for 325 ms (Figure 1A). At that time the animal would receive a liquid reward (R) or no reward (NR), depending on the trial type. If the NHP failed to correctly finish a trial, the same trial type would be repeated. For the manual task, the trial types were randomized otherwise. For the observation task, the trials were presented in a sequenced pattern alternating between R and NR trial types for NHP-Ac. For NHP-Zi the observational data was biased with a 2:1 ratio in favor of R trials, but otherwise randomized.

For the observational task (OT), the NHPs observed the hand feedback cursor as it automatically moved at a constant speed from the center to the peripheral target (Figure 1B). The KINARM was locked into place and the NHP’s left arm was restrained to prevent reaching movements. Eye-tracking was used to ensure that the NHP was looking at the computer projection in the horizontal plane on a semi-transparent mirror where the tasks were displayed during the OT trials.

For each trial, there were four defined task phases or period for analysis: pre-cue (500 ms), post-cue (500 ms), pre-reward (500 ms), and post-reward (500 ms).

### Surgery

Two bonnet macaques (*Macaca radiata*) were implanted in M1 with a 96-channel microelectrode array (10 × 10 arrays separated by ~400 μm, 1.5 mm electrode length, 400 kOhm impedance, ICS-96 connectors, Blackrock Microsystems) using techniques detailed in our previous work (Chhatbar et al., 2010). All surgical procedures were conducted in compliance with guidelines set forth by the National Institutes of Health Guide for the Care and Use of Laboratory Animals and were further approved by the State University of New York Downstate Institutional Animal Care and Use Committee.

NHPs were implanted with a head post (Crist Instrument) 3 to 6 months before the electrode implantation. Implantation was performed after animals were trained to complete the manual task (see below) with a success rate of 90%.

#### Data Collection

After 2–3 weeks of recovery, single-unit and multi-unit activity were recorded using multichannel acquisition processor systems (Plexon). The signals were amplified, bandpass filtered from 170 Hz to 8 kHz, and sampled at 40 kHz before the waveforms were sorted using principal component (PC) methods (Marsh et al., 2015). There was no segregation between single (SU) and multi-unit (MU) for our analysis, as the use case is towards brain-computer interface (BCI) applications where both SU and MU have been *shown* useful in providing information. For NHP-Ac (contralateral M1), data was taken from the contralateral M1 and the ipsilateral M1 for NHP-Zi (ipsilateral M1) concerning the right arm that was used in the reaching task.

For NHP-Ac manual task, there were four same-day recording sessions included in this analysis with 80, 80, 80, and 112 units detected after offline sorting from sessions 1, 2, 3, and 4 respectively. For NHP-Ac’s observation task, there were three same-day recording sessions included with 91 units recorded in each using the same MAP sort file (Plexon) indicating these may be the same units (SU and MU). The number of trials for NHP-Ac was *N* = 190 R and 61 NR for manual and 469 for both R and NR observational trials. The data collected from NHP-Ac OT was a perfectly predictable sequence of R trials followed by NR and repeating. This structure allowed the NHP to learn the trial value sequence as shown in Tarigoppula et al. (2018) with this same data. For Monkey *Z* manual task, there were three different-day recording sessions used for this analysis with 38, 36, and 32 units detected after offline sorting (Offline Sorter from Plexon). For the observation task, there were also three different-day recording sessions included with 42, 45, and 40 units recorded in each. The number of trials for NHP-Zi was *N* = 326 R and 159 NR for manual, and 519 R and 258 NR for observational data. It should be noted that the total number of units recorded from was most likely less than the full numbers above, especially for the same day sessions of NHP-Ac as many of the units may have been the same between sessions, however, for this analysis, we did not separate SU and MU data explicitly.

## Data Analysis

### Firing Rate

To determine differences between R and NR trials for each unit, firing rates were calculated using overlapping bins of 100 ms moving in increments of 5 ms. For each unit, *n*, the firing rate, $V_{n}^{k}(b)$, of each bin, *b*, of each trial, *k*, was determined by:

(1) $$V_{n}^{k}(b)=\frac{c_{n}^{k}(b)}{T}$$

where $c_{n}^{k}(b)$ is the number of spike counts per bin and *T* is the length of the bin in ms. For each unit, the firing rate was normalized across the entire session using min-max normalization on a per unit basis,

(2) $$V_{normalized_{n}^{k}}(b)=\frac{V_{n}^{k}(b)-\min(V_{n})}{\max(V_{n})-\min(V_{n})}$$

The normalized firing rate, $V_{normalized_{n}^{k}}(b)$, is distributed between 0 and 1. For certain analyses, the firing rate data was not normalized, such as when looking at the influence of rate on noise-correlation described below.

### Noise Correlation

Noise-correlation refers to the trial-to-trial variations that are shared between pairs of units (Cohen and Kohn, 2011). Noise-correlation is equivalent to the Pearson cross-correlation of the trial-by-trial firing rate variation of two units about their respective bin-wise means (Bair et al., 2001; Kohn and Smith, 2005). The trial-by-trial firing rate variation was calculated for each unit, *n*, by

(3) $$V_{noise_{n}^{k}}(b)=V_{normalized_{n}^{k}}(b)-\frac{\sum_{k=1}^{K}V_{normalized_{n}^{k}}(b)}{K}$$

where *K* was the total number of trials. The Pearson coefficient of *V*_noise_ was found by

(4) $$r_{noise}(n_{1},n_{2})=\frac{\text{cov}\left(V_{noise1},V_{noise2}\right)}{\sqrt{\text{var}}\left(V_{noise1}\right)\text{var}\left(V_{noise2}\right)}$$

and was calculated using the MATLAB function “corr.” The correlation was calculated separately for each trial period, and all trials of the same trial type were concatenated to find a single coefficient per unit pair per task phase.

### Population-Level Noise-Correlation Analysis

In addition to the above NC analysis conducted on unit pairs, we additionally utilized principal component analysis (PCA) to determine from the population of units a measure of the dimensionality of the dataset during R vs. NR trials. The hypothesis was that during NR trials there would be less variability in the data, as seen previously (An et al., 2019), and therefore the amount of variability from the full dataset accounted for by 20 PCs should be greater during NR trials as compared to R trials. PCA was run separately on the R and NR trials. We then determined the % variance accounted for and used that measure as our variable to compare.

### Rate vs. Noise-Correlation

The effect of firing rate on noise-correlation was quantified by analyzing the relationship between the arithmetic mean of the unit’s raw activity determined for R and NR data separately. The noise-correlation coefficients during the post-cue response period of all unit pairs were determined for R and NR data separately. Next, we determined which unit pairs from all units had mean absolute differences in their mean post-cue period rate less than 0.8 Hz, which was chosen such that for each data set there was data within each bin. We then took these pairs of units from the R and NR datasets and formed 10 bins, with each bin containing data with a mean individual unit rate within the 0.8 Hz bin. We then plotted this mean rate (*x*-axis) against the mean of the absolute value of the noise-correlation between the unit pairs within that rate bin. We ran a Wilcoxon Signed Rank test (signed-rank test in MATLAB set with “tail,” “left”) on the data as well as a Kolmogorov–Smirnov test (kstest2 in MATLAB). Both statistical tests were used as they are sensitive to different features, such as the KS test being more sensitive to any differences between the two distributions being compared, while the rank-sum test is more sensitive to differences in the medians. Also, we utilized the one-sided signed-rank test to address the hypothesis that NC was higher for NR trials as compared to R trials. We focused on the post-cue response period, which was consistently different between R and NR trials for all tasks and NHPs for the median noise correlations (Figure 4). Unit pairs from all sessions for a given NHP-Ac and task type were combined for this analysis (Figure 5). For individual session median noise correlations see Figure 4.

**Figure 2:**
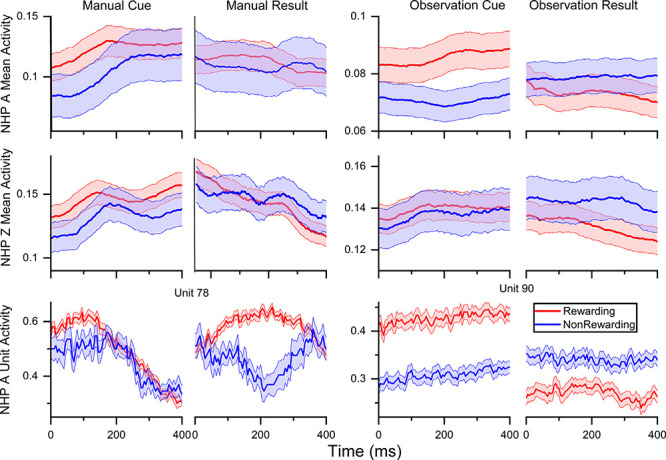
Normalized (min-max) mean firing rate ± SEM for rewarding (R, red) and non-rewarding (NR, blue) trials averaged across all units and sessions as follows, NHP-Ac manual, *N* = 80, 80, 112, 80 units across four sessions, NHP-Ac observational, *N* = 91 across all three sessions, NHP-Zi manual *N* = 38, 36, 32 units across three session and NHP-Zi observational *N* = 42, 35, 40 units across three sessions. In cue plots, zero is the time of the cue being shown, and in result columns, zero is the time the result started. Plotted data was binned at 100 ms and a moving window of 5 ms was used to smooth the data for presentation purposes.

**Figure 3:**
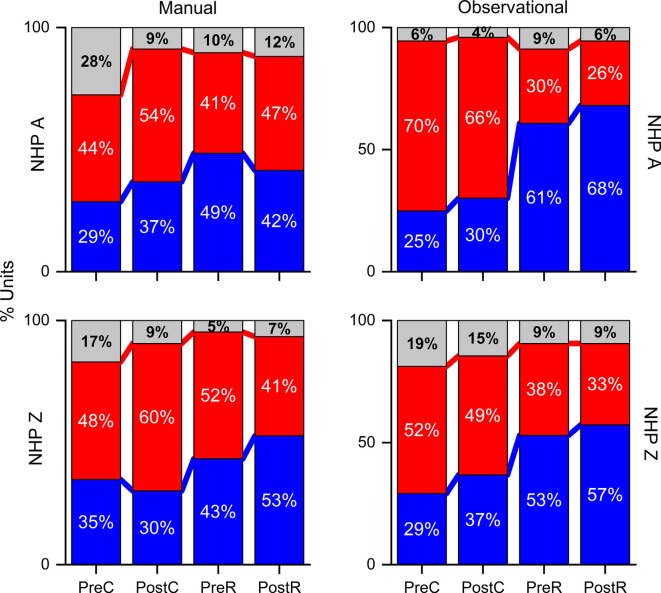
Percentage of units with significantly higher firing rates for R (red) and NR (blue) trials in each period, pre- post-cue, pre- and post-result, as well as the percentage of units that did not significantly reward level modulated. The number of units for each NHP-Ac and task are as shown in Figure 2 at approximately *N* = 272 NHP-Ac manual, 273 NHP-Ac observational, 100 NHP-Zi manual, and 117 NHP-Zi observational, see “Materials and Methods” section. There were three sessions for each NHP-Ac and task except for the NHP-Ac manual with four.

**Figure 4:**
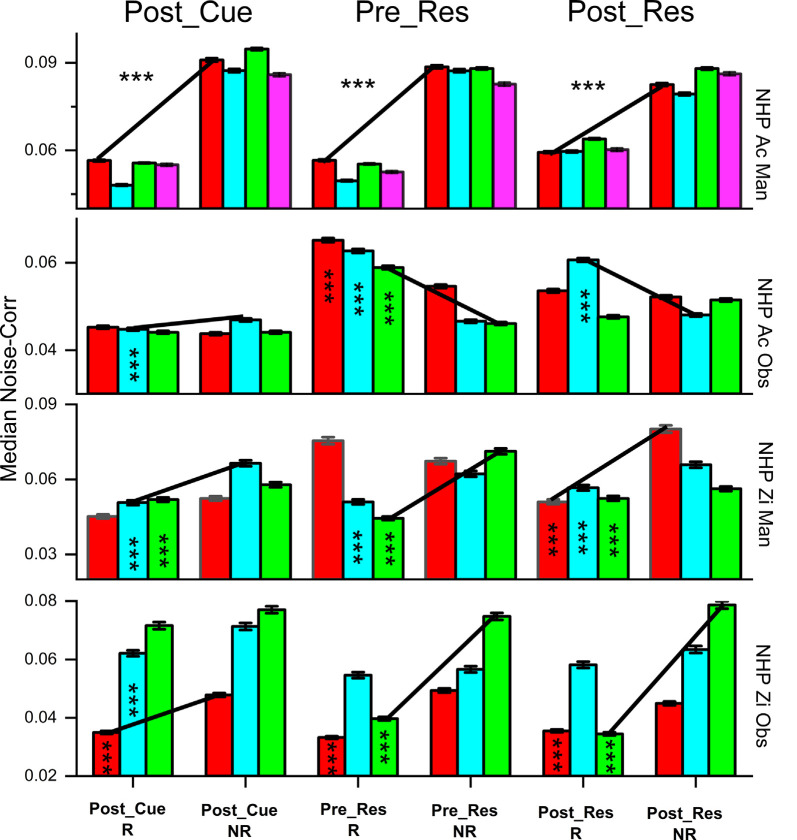
The median absolute value of noise correlations (NC) ± SEM, for R and NR trials during the post-cue, pre-result and post-result periods. Significant differences were determined by Wilcoxon rank-sum (*p* < 1 × 10^−10^***). All bars deemed significant for the rank-sum test were also significant utilizing the Kolmogorov–Smirnov test. The trial type is shown at the bottom and color indicates the data set # for the individual non-human primates (NHPs) in order from left to right (red dataset #1, cyan dataset #2, et cetera), see “Materials and Methods” section for more on these sets. The black lines are used to show the trend between the R and NR trial types. We have included the asterisks in the left bars for a pair being compared (R vs. NR).

**Figure 5:**
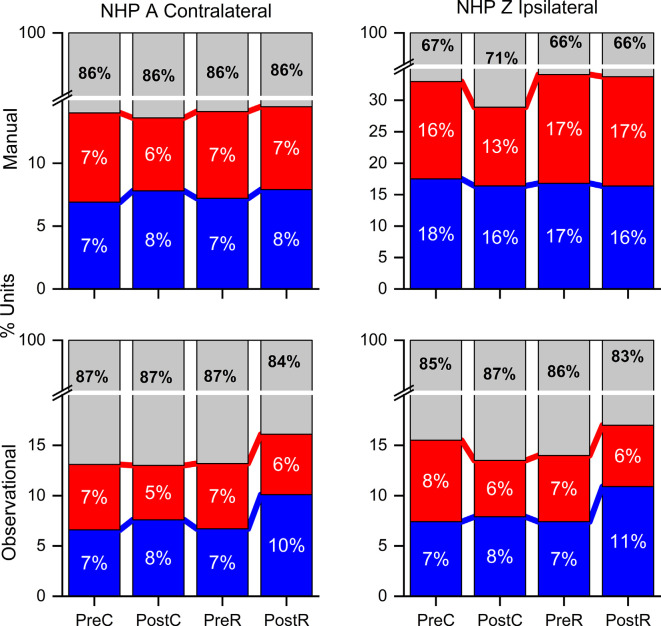
Percent of unit pairs that have higher noise-correlation for R (red), NR (blue), or neither (gray) trial type for both NHPs and tasks as labeled during the pre- and post-cue, pre- and post-result periods. Note the break in the *y*-axis.

### Relationship Between Signal and Noise Correlation

**Figure 6:**
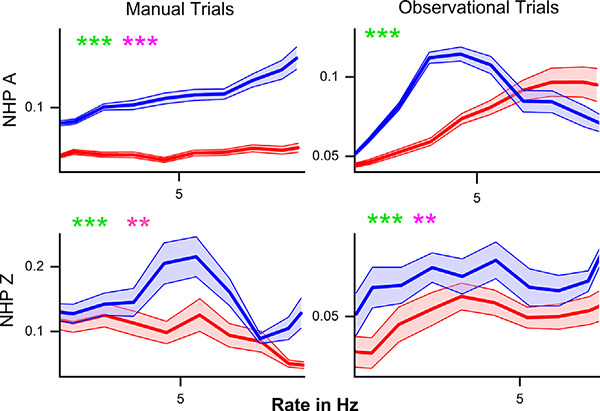
The mean noise-correlation coefficient (y-axis) plotted against the mean rate for unit pairs with similar mean rates (<0.8 Hz difference) during the post-cue period. R (red) and NR (blue) trials are plotted ± SEM for all unit pairs over all data sets for that NHP-Ac and task type. For each subplot, we have included statistical results from both the Kolmogorov–Smirnov test (green) as well as the Wilcoxon rank-sum test (pink) with **p < 0.01, and ***p < 0.001, between R and NR trials.

We conducted an analysis of covariance (ANCOVA) between the signal- and the noise-correlation for all unit pairs recorded during R and NR trials separately. We utilized the MATLAB function aoctool for this analysis with the model set to “separate lines,” such that there were no added constraints to the model. We have plotted for each unit pair the signal correlation (*x*-axis), which is simply the crosscorrelation between the mean unit responses (PSTH, post-stimulus-time-histogram) during the given task period, against the noise-correlation for the unit pair. The aoctool outputs statistical confidence on the model parameter values as well as an ANOVA table on the signal vs. noise, the trial type of R vs. NR, and interaction of Signal-correlation * Reward level vs. noise-correlation, and we have included the ANOVA outputs on the related figure, or accompanying text (Figure 6).

### Identifying Reward Modulated Subpopulations

Selecting reward discriminatory units was achieved using the Pearson correlation between reward value on a given trial and unit firing rate, where R was assigned a value of 1, and NR was assigned a value of 0. We arbitrarily used the top 20% of units with the highest correlation coefficients, showing a strong positive correlation with reward, which were classified as “High Positive” units. The bottom 20% of units with the lowest correlation coefficients, showing a strong negative correlation with reward, were classified as “High Negative” units. Last, the middle 20% of units within the middle of the distribution of absolute value correlation coefficients, were classified as “middle-responsive” units. These subpopulations were determined for each period separately, which means they may consist of different units during each period. This analysis was carried out to determine if all three types of units would still show modulation in their noise-correlation and if these populations would differ towards finding the best information channels on cortical state related to reward expectation, reward-associated motivational salience, or attention.

## Results

We present our results addressing our main hypothesis that reward expectation influences trial-to-trial noise-correlation, such that this measure could be used to track the cortical state of expecting reward vs. not expecting a reward, which we aim to use towards autonomously updating BCIs. Toward this aim, we recorded from neural ensembles simultaneously (30–112 units) in contralateral or ipsilateral M1 in NHPs performing a single target reaching task, or observing such a task, as seen in Figure 1. To present a fuller story we first show raw data that indicates reward expectation modulates M1 rates as we have previously described (Marsh et al., 2015; Tarigoppula et al., 2018; An et al., 2019). We start our results with raw PSTHs between R and NR trials (Figure 2), moving onto inquiry about the % of units that modulate their rate to reward-level cueing (Figure 3), after which we show the mean noise-correlation for each dataset for R and NR trials (Figure 4). We then looked at how the firing rate influences noise-correlation using mean matched methods (Figure 5) and then shows a correlational structure between the noise-correlation and the signal correlation (Figure 6). Finally, we look more closely at how subpopulations of units noise-correlation are modulated by reward expectation, such as units which have increased rate modulation due to reward, or decreased rate, or no reward modulation shown in rate, as well as how high positive correlated units are modulated by reward cueing vs. highly negatively correlated units.

In Figure 2, we have plotted the average min-max normalized mean firing rate by trial type, R (red) and NR (blue), across all units for all sessions. NHPs (A, contralateral, and Z, ipsilateral) and task types (manual and observational). These results align with previous work showing an increased firing rate in response to a preferred stimulus and a higher overall rate for R stimuli (Marsh et al., 2015; Tarigoppula et al., 2018) and utilized some of the same datasets used here from the same NHPs. This trend can be seen in both the manual and observation tasks, indicating that reward modulation occurs in the motor cortex while performing a reaching movement and during passive observation as expected (Marsh et al., 2015; An et al., 2019). Also, there are differences between the manual and OTs consistent with previous research, where neural responses to observation tend to be weaker than during the manual version of the task. We have plotted two example units for NHP-Ac manual and OTs in the bottom row of Figure 2. NHP-Ac’s observational data is separated between R and NR even at the start of the pre-cue period. This separability can be attributed to the predictable nature of the trial value sequence for NHP-Ac’s OT, which had a repeating sequence of R followed by NR trials, in comparison to the more randomized trial sequence in the manual task (see “Materials and Methods” section). As NHP-Ac learned the sequence of R-NR the difference in firing rate can be seen before the cue onset as shown in Tarigoppula et al. (2018) for these data sets. Please note results on the above datasets, and related datasets, for differences in firing rate, duration of trials, and EMG have been presented previously by us showing the expected results, such as slightly increased trial duration during manual tasks for NR trials as compared to R trials (Marsh et al., 2015; Tarigoppula et al., 2018; An et al., 2019; Hessburg et al., 2019). However, the OTs have no such differences, and as seen in our results still show clear differences due to reward level cuing.

Figure 3 illustrates the percentage of units in each period that were modulated by the cued reward level. In each graph, red indicates a higher firing rate for R-trials, blue indicates a higher firing rate for NR-trials, and gray indicates no significant difference between the two trial types (Wilcoxon rank-sum, *p* < 0.05). For both NHPs, most of the units were modulated for reward when the firing rate was compared in each period. There is a larger percentage of units with significantly higher firing rates for R trials in the post-cue period, and this percentage decreases as the trial phases move forward (Figure 3). During the pre-result and post-result periods of the OT, both NHPs show a greater percentage of units with an increased firing rate in NR trials as shown previously (Tarigoppula et al., 2018).

The differences in firing rate between R and NR trials confirm that reward modulation occurs in the motor cortex during these single target center-out reaching tasks (Marsh et al., 2015), which has also been shown in multi-target tasks (Ramkumar et al., 2016; Ramakrishnan et al., 2017; Tarigoppula et al., 2018; Zhao et al., 2018). This metric alone, however, does not provide a complete perspective on the dynamics of these populations. Further analyzing the interactions between these units is critical to understanding more broadly the cortical state.

### Noise Correlation

The observed noise-correlation (NC) includes both positive and negative coefficients which result in a mean NC close to zero, similar to the range given by Ecker et al. (2010) of 0.01–0.03. Figure 4 displays the absolute value of the unit pair NC for all unit pairs. The results show NC for R and NR trials, with some variation across periods, task, and subject. During the manual and OTs, as hypothesized, both subjects showed significantly higher NC for NR trials during the post-cue period. During the pre- and post-result period, both NHPs for the manual task show the same relationship of higher NC for NR trials and so did NHP-Zi for the OT. However, NHP-Ac, which had the predictable trial value sequence for the OT, showed the opposite relationship during the pre- and post-result periods as seen in the second row of Figure 4.

In addition to the above pair-wise NC analysis, we also conducted PCA at the population level of our datasets. PCA determines the eigen-vectors and values of the covariance matrix from the full population dataset, and we wished to know if we would see similar results to those from the pair-wise analysis, as we expected. In short, we did obtain the same qualitative trends. We found that the first 20 PCs from PCA run on NR trial data alone always accounted for more of the variance of the neural population data than when conducting PCA on R trial data alone. This result was expected due to the relationship between the pair-wise analysis, and the use of the covariance matrix in PCA, but allows us to show with clarity similar results for pairwise and population-level analysis.

### Percent of Population

Considering all unit pair noise-correlation coefficients, the significance of the difference between R and NR NC was determined by the Wilcoxon rank-sum (*p*-value < 0.05) for each unit pair. Unit pairs were classified as: either having a higher NC during R (red) or during NR trials (blue; Figure 5). NHP-Ac showed a slightly greater percentage of unit-pairs with higher NR NC in most periods. A larger percentage of unit pairs do not demonstrate a significant difference between R and NR trials for their NC (gray), suggesting that NC differences on the Unit level are not strong and the population as a whole carries this information (compare Figures 4, 5). NHP-Ac observation, NHP-Zi manual, and NHP-Zi observation data all follow a similar trend with slightly more unit pairs showing higher NC for NR trials (Figure 5). The post-cue period consistently showed a slightly higher percentage of unit pairs with increased NC for NR trials across all NHPs and tasks.

### Effect of Firing Rate

Previous work indicated that an increase in firing rate causes an increase in NC (de la Rocha et al., 2007). Both NHP-Ac and Zi demonstrate higher NC for NR trials regardless of the relationship between post-cue induced firing rate changes and NC. The relationship between mean NC and mean matched firing rates differed between the trial types and NHPs (Figure 6). In NHP-Ac, NC showed a linear increase with the firing rate during the manual task for NR trials. However, for the observation task, there was an inverse effect on NR trials that resulted in a sharp decrease in NC at higher firing rates, where data from R trials showed a more linear increase in NC with firing rate (Figure 6). For NHP-Zi, there was a significant tendency for NR trials to have higher NC for all rates as seen in Figure 6. However, the relationship between rate and NC was not simply linear, as also seen for NHP-Ac observational data. These relationships remained whether we used the geometric mean (data not shown) or the arithmetic mean. In Figure 6, green asterisks are used to report results from KS-tests, while the pink asterisks are results from Wilcoxon signed-rank tests. In Figure 6, we focus on the lower rage of firing rates as R trials, as seen in Figure 2, had higher rates and NR trials would have very few or no rate bins with data in them at those higher rates. However, when using a geometric mean binning method and spanning the full rate space we did not see qualitatively different outcomes (data not shown).

### Signal vs. Noise Correlation

**Figure 7:**
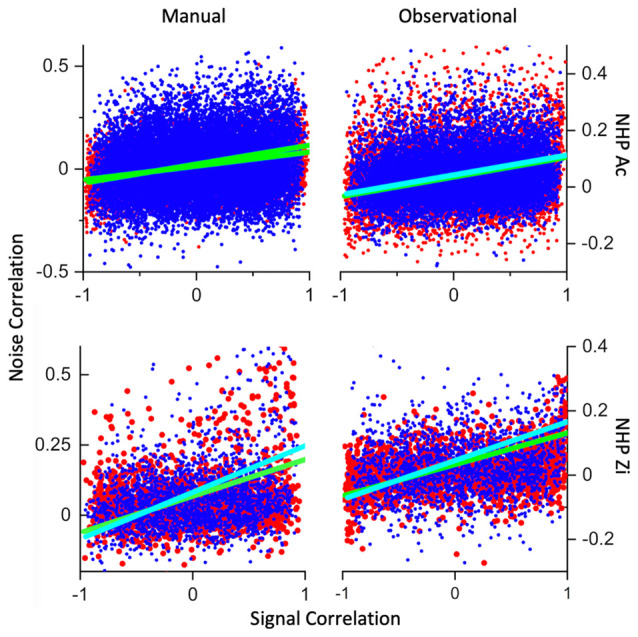
Signal- vs. noise-correlation. Plotted in red are the R trial datapoints from all datasets for each NHP and task, while blue datapoints are from the NR trials. Cyan line is a linear fit to the NR trials and green are linear fits to the R data. The x-axis is signal-correlation while the y-axis is the noise-correlation. All datasets had highly significant linear fits between most of the variables involved, including trial-type, y-intercept and slope of the linear fits. All datasets for NHP-Zi, and the manual datasets for NHP-Ac also showed highly significant fits for signal-correlation *trial-type vs. noise-correlation. Statistics included below from the ANOVA output for the relationships between signal-correlation, noise-correlation and trial-type. MATLAB aoctool was utilized for this analysis of covariance (ANCOVA). Number of data sets was (*N* = 3 for all but NHP-Ac Manual *N* = 4, see “Materials and Methods” section for trial and unit #s). NHP-Ac manual task, group (R vs. NR), *p* = 0, *F*_(1,61,743)_ = 63, group * X (signal correlation) *p* = 0, *F*_(1,61,743)_ = 100. NHP-Ac OT, group, *p* = 0, *F*_(1,49,682)_ = 35. NHP-Zi manual task group *p* = 0.0004, *F*_(17,524)_ = 13, group * X *p* = 0.001, *F*_(17,524)_ = 11. NHP-Zi OT group *p* = 0, *F*_(19,016)_ = 17, group * X *F*_(19,016)_ = 23, *p* = 0.

Previous work has shown a relationship between the signal-correlation and the NC, and we wished to determine if the reward expectation level would influence this signal- and noise-correlation relationship. We have plotted the signal correlation for every unit pair for the R and NR trials as red and blue points in scatter plots as seen in Figure 7, along with linear fits to both data types separately. In both NHPs and tasks there were significant linear model parameters for intercept and slope (R vs. NR) Prob > |T| = 0. In addition, both NHPs for manual data, and NHP-Zi for observational, had significant differences between signal-correlation * trial-type vs. noise-correlation, NHP-Ac manual task, *p* = 0, *F*_(1,61,743)_ = 100, NHP-Zi manual task *p* = 0.001, *F*_(17,524)_ = 11, and NHP-Zi OT *F*_(19,016)_ = 23, *p* = 0. However, NHP-Ac’s observational data did not show this relationship between group and signal- vs. noise-correlation significantly, only for trial-type *p* = 0, *F*_(1,49,682)_ = 35, and signal- vs. noise-correlation *p* = 0, *F*_(1,49,682)_ = 3271 without regard to trial type that is group.

### Sign (+/−) of Noise-Correlation and Stability

**Figure 8:**
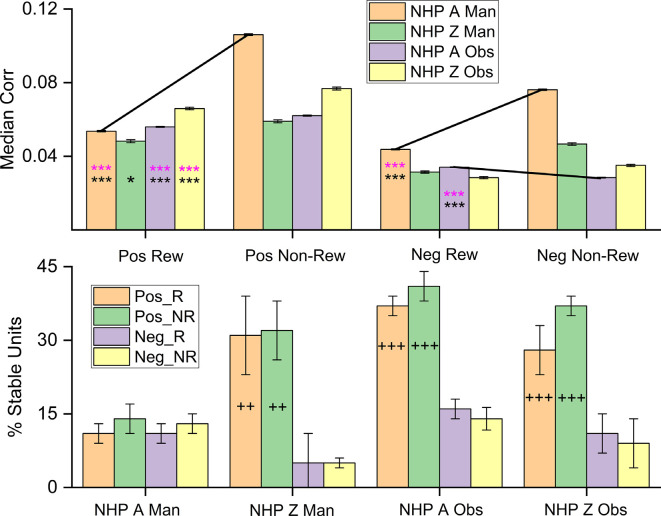
Median noise-correlation during the post-cue period for positive (Pos) and the absolute value of the negative (Neg) noise-correlation coefficients during R (Rew) and NR (Non-Rew) trials from all data (Top Row, ± SEM, for the median, over all data points from all dataset). Significance was determined using KS-test and Wilcoxon signed-rank) *,***, where *p* < 0.05, *p* < 0.001 respectively). All comparisons between Pos data sets for R and NR trials were significant, where significance was determined by Wilcoxon rank-sum (*p* < 0.05) and KS-tests (*p* < 0.05), as were differences between Pos and the absolute value of the Neg trials (all *p* < 0.001). The standard error of the medians was on the order of 0.0004–0.0008 for the median plots. (Bottom Row) The percentage of units that remain within a single category (Pos_R, Pos_NR, Neg_R, Neg_NR) throughout the task from post-cue, during pre-results and post-result periods (± SEM over dataset percentages). The Percent stable units were significantly different after *post hoc* testing between the groups with + on their bar. The number of + signs indicates how many out of the three datasets showed *post hoc* differences between the Pos and Neg subsets for both R and NR trial types (*p* < 0.05 *post hoc*, MATLAB multicomparewith Tukey’s honest significant difference criterion). Note separate keys for top and bottom rows.

Figure 8 shows the separation of the median positive and negative NC for each NHP-Ac and task during the post-cue period (Figure 8, top row). Positive (Pos) and negative (Neg) NC is shown for both R and NR trial types. It is clear that the left two columns of each group of four bars, which are for the positive NCs, are higher than the negative two NC bar values seen in the right two columns of each group of four bars. Likewise, NC is generally higher during the NR trials as compared to the R trials. Previous studies have related lower information encoding capacity to increases in positive NC, referring to the mean response of a population of neurons (Zohary et al., 1994). Dissecting the distribution of these coefficients by separating positive and negative correlations may explain discrepancies between subjects and task types and reveal additional information about the role of NC (Chelaru and Dragoi, 2016). For NHP-Ac manual task, the positive and negative coefficients are both greater in absolute value for NR trials during all periods within the trials. This aligns with the mean and median NC, which are higher for NR trials (Figure 4 and related text). In general, the other periods within the task also show greater NC during NR trials for both positive and negative coefficients, and NHP-Zi OT displays similar trends to NHP-Ac manual data during all periods (data not shown).

The bottom row of Figure 8 shows the stability of the population of units within a given group, such as units with positive NC during R trials, positive NC during NR trials, negative NC during R trials, and negative NC during NR trials. In general, one can see that the positive units are more stable in the sense that they do not flip back and forth between having positive NC during the different task phase, but remain positive in their unit-pair NC, whether during R or NR trial types. This stability was significant for all datasets except for the NHP-Ac manual, which was unexpected when compared to the previous results on NHP-Ac manual data set. NHP-Ac was implanted in the M1 contralateral to the reaching arm and therefore during manual trials, this cortical region was most in direct control of the reaching movements, and NC may indicate a decrease in information capacity, this might explain these results. Nevertheless, NHP-Ac manual data still had the same overall trends as the other datasets with R trials being lower than NR trials.

### Relationship Between Reward-Rate Modulation and NC

**Figure 9:**
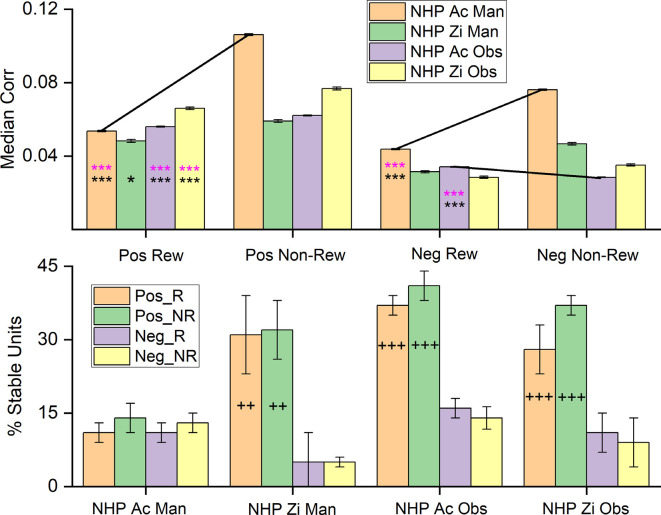
The median correlation coefficient for the High Positive, High Negative, and Low-responsive subpopulations each NHP-Ac and task as labeled. Black and pink asterisks located on the graph indicate there is a significant difference between the subpopulations during R and NR trials for Wilcoxon rank-sum and KStest2 respectively (*p* < 0.05, and *p* < 0.001 denoted by * and *** respectively).

While the combined neural firing rate of all recorded units shows separability between R and NR trials, there is evidence that this separability can be attributed to select units (Marsh et al., 2015). Analysis of the currently presented data sets revealed a subpopulation with an increased firing rate during R trials and another with an increased firing rate during NR trials (Marsh et al., 2015), which led directly to our Figure 9 analysis.

While it is apparent that NC differences in response to reward level cueing are present in only a subset of unit pairs (Figure 5), the defining features of these subpopulations are still unclear. To use noise-correlations as an indicator of reward expectation, selecting subpopulations that are known to modulate for a reward through firing rate provides a more direct comparison between these two factors. Figure 9 shows the median absolute value of the NC for R and NR trials for each of the following subpopulations: 1. units with rates that are upmodulated during R trials (High Positive), units with rates that are downmodulated during R trials (High Negative) and units not modulated strongly by reward (Low). NHP-Ac’s contralateral M1 showed the strongest relationship during the manual task with NR trials having higher NC for all subpopulations, and this was also seen for the high positive subpopulation for NHP-Ac’s observational data. Though NHP-Zi’s ipsilateral cortex showed little of this clear relationship, however, NHP-Zi’s data still maintained an overall trend of higher magnitude NC during NR trials compared to R trials (Figure 9).

## Discussion

The primary goal of this analysis was to determine if significant differences in NC exist between cued R and NR trials, and if so, to what extent might these differences relate to reward modulation of firing rates. As previously described, units with firing rates modulated by reward expectation are present in these data sets (Marsh et al., 2015; Tarigoppula et al., 2018), with one subset of M1 units demonstrating increased firing rates during R trials and one subset showing increased rate during NR trials (Figure 3). Because of the capacity for reward modulation demonstrated by these M1 units during both manual and observation tasks, it was hypothesized that there would be significant differences in NC between R and NR trials. Based on our published LFP work on increased levels of PAC and SFC in the alpha band during NR trials as compared to R trials (An et al., 2018, 2019), we expected to see increases in NC for NR compared to R trials as well. Recent work on these measures (PAC, SFC, and NC) in the frontal eye fields have suggested that NC modulations are due to long-range connections *via* LFP influences (Hassen et al., 2019).

Our presented results follow what would be expected if information capacity within the M1 network was governing the changes in neural rate, and correlational structure. To be clear, we hypothesize the M1 network is carrying the most information about the environment during the task cue period, when the NHP subjects learn the value of the given trial, as well as having information on the cursor position, velocity, and the target, etc. During this post-cue period, both the contralateral and ipsilateral M1s showed an increase in firing rates during R vs. NR trials (Figure 2). Corresponding to this increase in rate was a decrease in the median NC for R trials during the post-cue period (Figure 4), and an increase in NC for the NR trials. A potential factor impacting increased NR NC is the firing rate. Previous studies have demonstrated that increases in firing rate are related to increases in correlation (de la Rocha et al., 2007). However, we see the opposite influence of NR on the firing rate, that is NR decreases the firing rate on average. “Integrate-and-fire” models indicate that output correlation increases in response to increasing firing rate when the input correlation remains unchanged (de la Rocha et al., 2007). This pattern is illustrated by the increase in NC with increasing response firing rates seen in (Figure 6). However, despite the relationship with the firing rate, NR trials continue to exhibit higher NC than R trials. The response to changes in R stimuli is similar to the pattern seen in attention, with higher evoked rates leading to greater differences between the two states (Cohen and Maunsell, 2009). With increases in response to the value cue, the difference between NR and R NC increased in general, but not all responses were linear, and some decreased after reaching a given rate (Figure 6), and again, NR trials had lower firing rates than R trials. Thus, the increased rate for R trails is accompanied by a decrease in NC, a situation that could allow for more information-carrying capacity vs. the NR trials.

In addition to studying the pair-wise correlational structure, we also conducted Principal Component (PC) Analysis (PCA) on our data, finding the same trends seen in Figure 4 between R and NR trial type. Using PCA we asked when the latent dimensionality of the data was lower, during R or NR trials. More of the data’s variance was consistently explained by 20 PCs for NR trials than for R trials, again indicating that there is more correlational structure during the NR trails, leading to a lower-dimensional latent space, as compared to the R trials (data not shown). This increased correlation and decreased firing rate during NR trials, shown above, could indicate a cortical state with less information-carrying capacity, perhaps as a strategy to conserve energy during low reward expectation moments, which would likely correspond to states of low motivation and decreased attention, this is purely speculative though. By information-carrying capacity, we mean in the information-theoretic sense, where correlation decreases the capacity for a network to transmit information. One can imagine if all the neurons were perfectly correlated it would be similar to having just one neuron, whereas if they are all uncorrelated each neuron could be transmitting independent information at the same time (see for review or introduction respectively; Rieke et al., 1996; Magri et al., 2009).

Several possible explanations underlie the increase in NC during the post-cue period of NR trials including changes in attention, firing rate, motivation, and reward expectation. The first relies on changes in visual attention, where attention may either be a coexisting, or driving factor of correlation, or a separable, but resembling factor that produces similar dynamics to those induced by reward or reward-related motivation. Given the predictable nature of the trial reward value sequence (NHP-Ac observation), visual attention could be a confounding factor when discussing the relationship between reward and NC. It is possible that the subjects’ ability to anticipate a lack of reward resulted in a shift to an unattended state during NR trials, even before the trial started. Unattended trials show increases in correlation (Cohen and Maunsell, 2009; Mitchell et al., 2009; Herrero et al., 2013) and could account for the higher correlation coefficients seen in the NR trials. We have shown previously that using a task with multiple levels of reward leads to both linear trends in firing rate with value (Tarigoppula et al., 2018), as well as non-linear gain modulated activity patterns (Hessburg et al., 2019), making it seem unlikely that all of our results are attributed to attention alone unless the attention is modulated as one would expect the state-value to be modulated, or the state-motivation. However, further analysis and more directed experiments are needed to convincingly address these questions.

Attention, stimulus contrast (Kohn and Smith, 2005), learning (Gu et al., 2011; Jeanne et al., 2013), and global cognitive factors (Ruff and Cohen, 2014) have been shown to decrease NC in neurons within the same cortical area. Given the wide variety of factors impacting correlation, the increases during the NR trials could be the result of the independent modulatory influence of reward on NC. Previous studies provide insight into how this difference in correlation between states serves to improve information encoding. High levels of positive correlation can be detrimental to the coding accuracy of a similarly tuned population of neurons (Zohary et al., 1994). Cohen and Maunsell (2009) determined that the “modulation of noise-correlation accounts for the majority of the attentional improvement in population sensitivity,” with modulation of firing rate and single unit variabilities accounting for a much smaller portion of the change. Similarly, high correlation limits signal-to-noise ratios (SNR), with higher correlations leading to lower saturation of SNR as a function of neuronal pool size (Mitchell et al., 2009). Much like the trends seen in this research, Mitchell et al. (2009) noted that the effects of firing rate on the SNR saturation point do not adequately account for the differences in SNR ratios observed between units with high or low correlation.

There is evidence that increases in NR trial NC are related to reward modulation. However, the mean correlation does not provide a complete picture of the correlation activity in the population. An additional factor that may be impacting reward modulation is the distribution of positive and negative correlation coefficients. A mean positive correlation reduces the coding capacity of similarly tuned neurons by reducing the beneficial effects of adding or averaging responses, a limitation not present with units showing negative correlation (Zohary et al., 1994). More unit pairs share a positive correlation and the change in the positive correlation coefficients between NR and R trials shows a greater impact on the mean correlation. This aligns with previous evidence that changes in positive correlation specifically are the primary cause of the overall change in mean correlation. In the visual cortex, orientation changes have been found to decrease positive correlation while negative correlation remained stable with shifts in the preferred direction (Chelaru and Dragoi, 2016). In a recurrent network model, a lower firing rate and decreased negative NC is generated with increased local inhibition and reduced excitation, resulting in an increased SNR (Chelaru and Dragoi, 2016). However, our results lean in the direction of decreased SNR as NC increases with a decreased firing rate for our data as NR trials had both a decreased overall rate and an increased NC.

Our results show that R trials have decreased positive NC and a negative NC that most often either decreases or remains unchanged (Figure 8). The combined effect typically results in a decrease in median NC, indicating perhaps more information encoding capacity. Our results could be adhering to the trends seen in tuning direction, where the negative correlation is unchanged in response to changes in condition.

Our results support the hypothesis that NR trials should have higher NC coefficients. The lower overall correlation values during R trials align with previous research suggesting that decreases in correlation correspond with increased encoding capacity is similarly tuned neurons and homogeneous populations. Based on this, it is possible to conclude that NC plays a role in reward modulation in the motor cortex. Moving forward, this work has applications for the improvement of BCIs. Incorporating the context-dependent changes in NC into neural decoding models may improve decoding efficiency and increase BCI performance, as we have shown utilizing a neural critic based on classifiers of neural rate and LFP-PAC (An et al., 2018; Zhao et al., 2018). Likewise, incorporating NC into the neural state representation for a neural critic to be used in a RL BCI should help improve the performance of such RL-BCIs, where the neural critic would track the user’s brain state related to reward expectation and reward prediction error and be used to autonomously update the BCI to perform better for the user (Sanchez et al., 2011; Prins et al., 2017; An et al., 2018), or inform the decoder/policy (Zhao et al., 2018).

## Data Availability Statement

The datasets generated for this study are available on request to the corresponding author.

## Ethics Statement

The animal study was reviewed and approved by State University of New York Downstate Medical School IACUC.

## Author Contributions

BM performed the data analysis and prepared the manuscript. JF supervised and participated in all aspects of this research. All authors contributed to the article and approved the submitted version.

## Conflict of Interest

The authors declare that the research was conducted in the absence of any commercial or financial relationships that could be construed as a potential conflict of interest.
